# Supplementary material for: APEX2-Mediated Proximity Labeling Resolves the DDIT4-Interacting Proteome
Source: Int J Mol Sci. 2022 May 6;23(9):5189. doi: 10.3390/ijms23095189 (PMC9102673; doi:10.3390/ijms23095189)
Supplement: Supplementary file 1 [file ijms-23-05189-s001.zip › Suppl. Table 2.pdf]

Table S2. Primer sequences.

Primer set	Forward	Reverse
hs_DDIT4_CDS_for_APEX2_in_AAV_Rigid	ATCATTTTGGCAAAGAATTGGATCCACCGCCACCATGCCTAGCCTTTGGGAC	TGCTTCTGCGCCACCACACTCCTCAATGAGCAGC
APEX2_CDS_for_DDIT4_in_AAV_Rigid	TGAGGAGTGTGGTGGCGCAGAAGCAGCAGCAAAAGAAGCAGCAGCAAAAGCAGCACCGGGAAAGTCTTACCCAACTGTG	CCAGAGGTTGATTATCGATAAGCTTCTATTAGGCATCAGCAAACCCAAG
hs_DDIT4_CDS_for_APEX2_in_AAV_Flexible	ATCATTTTGGCAAAGAATTGGATCCACCGCCACCATGCCTAGCCTTTGGGAC	TGAACCGCCTCCACCACACTCCTCAATGAGCAGC
APEX2_CDS_for_DDIT4_in_AAV_Flexible	TGAGGAGTGTGGTGGAGGCGGTTCAGGCGGAGGTGGCTCTGGCGGTGGCGGATCGGGAAAGTCTTACCCAACTGTG	CCAGAGGTTGATTATCGATAAGCTTCTATTAGGCATCAGCAAACCCAAG
FLAG_APEX2_NES_for_AAV	ATCATTTTGGCAAAGAATTGGATCCACCGCCACCATGGACTAC	CCAGAGGTTGATTATCGATAAGCTTCTATTAGTCCAGGGTCAGGC
mmu_ DDIT4_CDS3’	ATATGGTACCGCCACCATGCCTAGCCTCTGGGATCGT	ATATGCGGCCGCACATGTTTATTAGAAAAGTA
